# Editorial: Deleterious and beneficial humoral immune response in viral diseases: Two sides of the same coin

**DOI:** 10.3389/fimmu.2023.1185852

**Published:** 2023-03-31

**Authors:** Feng-Liang Liu, Yuejin Liang, Pengfei Wang, Yong-Tang Zheng

**Affiliations:** ^1^ Key Laboratory of Animal Models and Human Disease Mechanisms of the Chinese Academy of Sciences/Key Laboratory of Bioactive Peptides of Yunnan Province, KIZ-CUHK Joint Laboratory of Bioresources and Molecular Research in Common Diseases, Kunming Institute of Zoology, Chinese Academy of Sciences, Kunming, Yunnan, China; ^2^ Department of Microbiology and Immunology, University of Texas Medical Branch, Galveston, TX, United States; ^3^ State Key Laboratory of Genetic Engineering, MOE Engineering Research Center of Gene Technology, School of Life Sciences, Fudan University, Shanghai, China

**Keywords:** antibodies, viral diseases, humoral immune response, animal models, vaccine, adjuvants, antigen (Ag)

The humoral immune response is a critical component of the immune system, responsible for defending humans and animals against diseases, while antibodies are the major arm of humoral immunity to fight against diseases. During the COVID-19 epidemic, several laboratories and companies worldwide have dedicated efforts towards the development of therapeutic antibodies. To date, at least 400 antibodies have been submitted to the Coronavirus Immunotherapy Consortium (CoVIC) (1).

The production of antibodies includes many steps: preparation of antigens, isolation of antibody candidates, *in vivo* or *in vitro* antibody generation, and evaluation of antibody candidates (including evaluation using animal models and clinical evaluation). There are a few obstacles in the design of antigens due to complex epitope structures. Miller et al. reviewed the recent progress in epitope complexity, encompassing topics such as epitope biochemical complexity, antigen conformational/dynamic complexity, as well as epistatic and allosteric complexity. To obtain more epitopes, Kar et al. constructed nanoparticle fusion antigens that fuse trimeric influenza stem domain antigen, pH1HA10, to the C termini of MsDps2 or Encapsulin, or the N terminus of Ferritin. Immunization of mice with nanoparticle fusion antigens showed that all antigens could protect mice against homologous and heterologous influenza virus infections, and the nanoparticle fusion antigens exhibited heightened protective effects compared with trimeric influenza stem domain antigens.

Protein antigen alone is insufficient in eliciting robust immune responses, and adjuvants are commonly used to help produce higher levels of antibodies and T-cell responses in humans and animals. dos-Santos et al. studied the effect of a combination of Alum and poly(I:C) on immune responses. As a result, the combination not only increase the production of anti-SARS-CoV-2 spike antibodies and neutralizing titers against SARS-CoV-2, but also induced higher levels of T-cell responses. Apart from being identified in immunized individuals, antibody candidates can also be discovered in infected humans or animals. Oliveira-Filho et al. reported the SARS-CoV-2 prevalence among domestic cats in Brazil. Their study also revealed that cats can generate antibodies to suppress SARS-CoV-2 infection, which may be a source of anti-SARS-CoV-2 antibodies. A single antigen could stimulate the production of multiple antibody types, so it is necessary to use a good strategy or technique to isolate monoclonal antibodies binding target antigens. Sun et al. employed the phage display technique to construct a large size of human antibody heavy chain variable (VH) domain library, which would be a benefit for screening antigen-specific antibodies. Using a similar phage library, Wu et al. had previously discovered three nanobodies targeting the SARS-CoV-2 receptor-binding domain (RBD) (2). In the Research Topic, they further found that one previously discovered nanobody, Nb22, showed broadly neutralizing activity against SARS-CoV-2, and when administered intranasally, it could inhibit SARS-CoV-2 replication in hACE2 mice.

Animal models are important tools to evaluate the efficacy of antibodies. SARS-CoV-2 is unable to bind mouse ACE2, which has led researchers to generate SARS-CoV-2 mouse models using human ACE2-transgenic mice (3). Yan et al. generated two lethal mouse-adapted SARS-CoV-2 (BMA8 and C57MA14), which could infect wild-type mice *via* mouse ACE2. The new virus could highly replicate in the upper and lower respiratory tract, resulting in severe disease and animal death. Therefore, BMA8 and C57MA14 are valuable tools used to evaluate antibodies in mice.

Antibodies, much like coins, have two sides. Except for benefits, they can also be detrimental in certain situations. Zang et al. reported a clinical case: one female patient infected with Japanese encephalitis virus (JEV) didn’t completely recover after antiviral, glucocorticoid, and immunoglobulin treatment. She showed a high level of antibody in blood and IgG in the cerebrospinal fluid leak (CSF). Protein A immunoadsorption (PAIA) therapy was used to decrease circulating antibodies. The patient slowly recovered as circulating antibodies decreased, suggesting that elevated antibodies are detrimental to this patient.

In conclusion, the original research articles and reviews in this Research Topic highlight the dual nature of the humoral immune response. While antibodies could be used to treat certain diseases, they can also have negative effects and contribute to the development of other diseases.

## Author contributions

All authors made a substantial, direct, and intellectual contribution to this work and approved it for publication.
